# Comparative evaluation of the vertical fracture resistance of endodontically treated roots filled with Gutta-percha and Resilon: a meta-analysis of in vitro studies

**DOI:** 10.1186/s12903-018-0571-x

**Published:** 2018-06-13

**Authors:** Minmin Tan, Zhaowu Chai, Chengjun Sun, Bo Hu, Xiang Gao, Yunjia Chen, Jinlin Song

**Affiliations:** 1 College of Stomatology, Chongqing Medical University, Chongqing, China; 2 Chongqing Key Laboratory of Oral Diseases and Biomedical Sciences, College of Stomatology, Chongqing Medical University, Chongqing, China; 3 Chongqing Municipal Key Laboratory of Oral Biomedical Engineering of Higher Education, College of Stomatology, Chongqing Medical University, Chongqing, China; 4 Stomatological Hospital affiliated to Chongqing Medical University, No. 426, N. Songshi Rd, Chongqing, 401147 China

**Keywords:** Gutta-percha, Resilon sealer, Root canal obturation, Tooth fracture, Meta-analysis

## Abstract

**Background:**

Teeth treated endodontically are more susceptible to vertical root fracture (VRF). Some studies have suggested that obturating the root canals with Gutta-percha or Resilon can reinforce endodontically treated teeth, but a few others have presented conflicting results. These inconsistent results cannot guide clinicians in determining clinical approaches. The objective of this meta-analysis is to evaluate and compare the vertical fracture resistance of endodontically treated root canals obturated with Gutta-percha/AH plus and the Resilon system.

**Methods:**

Comprehensive literature searches were performed in the PubMed, Cochrane Library, ScienceDirect, Web of Science and Embase databases. The titles and abstracts of all of the retrieved articles were independently assessed by two authors according to predefined selection criteria. Data in the included articles were independently extracted. Statistical analyses were conducted using Review Manager 5.3 and Stata 12.0 software. The pooled standardized mean differences (SMDs) with 95% confidence intervals (CIs) were calculated for the outcome indicators. The level of statistical significance was set at *p* < 0.05. The Cochran Q test (I^2^ test) was used to test for heterogeneity among studies.

**Results:**

Fourteen randomized controlled in vitro trials were included in the meta-analysis. The results demonstrated that the vertical root fracture resistance of unprepared and unfilled roots was significantly higher than that of roots obturated with Gutta-percha/AH plus (SMD = − 0.69, 95% CI = − 1.34 to − 0.04, *p* = 0.04) or the Resilon system (SMD = − 0.54, 95% CI = − 1.07 to − 0.00, *p* = 0.05). The differences in fracture resistance between the roots filled with Gutta-percha/AH plus and the prepared unfilled root canals was not significant (SMD = 0.59, 95% CI = − 0.02 to 1.21, *p* = 0.06). Roots obturated with Resilon had higher fracture resistance than instrumented unfilled roots (SMD = 0.83, 95% CI = 0.44 to 1.22, *p* < 0.0001) or roots filled with Gutta-percha/AH plus (SMD = 0.62, 95% CI = 0.01 to 1.23, *p* = 0.05).

**Conclusions:**

The present study suggests that filling with Gutta-percha/AH plus dose not reinforce endodontically treated roots, whereas obturating with the Resilon system can increase vertical root fracture resistance of prepared roots. As this meta-analysis was based on in vitro studies, it should be careful to extrapolate its conclusion to the clinical context.

## Background

Endodontic therapy is a commonly used approach for the treatment of pulpitis and periapical periodontitis. Endodontically treated teeth have been demonstrated to be more prone to crown or root fracture than vital teeth [1, 2]. Vertical root fracture (VRF) is the most common and serious complication of the endodontically treated tooth, which typically leads to root resection and tooth extraction [3]. Therefore, the prevention of VRF is desirable. Many attempts have been made to increase the strength of endodontically treated roots, such as placing posts inside the roots [4, 5] and obturating dental materials in the root canals [6–8]. In recent years, the effects of different obturating materials on the strength of endodontically treated roots have received substantial attention.

Gutta-percha with the resin-based sealer AH plus is regarded as the gold standard in current obturation systems. Although Gutta-percha has many excellent properties, including good biocompatibility, low cytotoxicity [9], dimension stability and thermoplasticity, the ability of this material to strengthen roots that are treated endodontically remains unclear. Some studies [2, 10–12] reported that Gutta-percha/AH plus significantly increased the VRF resistance of instrumented roots, whereas other studies reported no significant effect [13–19]. Resilon, a root canal obturating material based on thermoplastic-filled polymer composites, has excellent sealing ability, antimicrobial activity, adhesive properties and retreatable properties [20, 21] when used in combination with one of the dual-cure resin-based root canal sealers Epiphany or Realseal. However, whether the fracture resistance of root canals can be increased by filling with Resilon and whether roots obturated with Resilon have higher fracture resistance than do those filled with Gutta-percha remain unclear. The inconsistent results cannot provide clear guidance to clinicians in making appropriate clinical choices.

Therefore, it is imperative to conduct a meta-analysis to investigate and compare the strengthening effects of Gutta-percha and Resilon on prepared roots. For the purposes of the meta-analysis performed here, data from randomized controlled in vitro trials were compiled to evaluate and compare the effects of these two root canal filling materials on the VRF resistance of teeth after root canal therapy.

## Methods

### Search strategies

Comprehensive searches of the relevant literature were performed in the PubMed, Cochrane Library, ScienceDirect, Web of Science and Embase databases from the earliest available date to November 21, 2017. The main keywords used were ‘gutta-percha’, ‘resilon sealer’ and ‘tooth fracture’. For instance, the free search terms used in PubMed were as follows ‘(((gutta-percha) AND (AH plus)) OR (resilon)) AND ((tooth fracture) OR (fracture resistance))’. Specific searching strategies were developed for each database, as shown in Table 1. Additionally, the references in each retrieved articles were evaluated to avoid the omission of any relevant articles.

**Table 1:** Database search strategy

Pubmed	1. “Gutta-percha” [all fields] OR “Guttapercha” [all fields] 2. “AH-plus” [all fields] OR “Epoxy Resins” [all fields] OR “Resin Cements” [all fields] OR “epoxy resin-based root canal sealer” [all fields] 3. “Resilon” [all fields] 4. “tooth fracture” [all fields] OR “vertical fracture” [all fields] OR “fracture resistance” [all fields] 5. “english” [language] 6. (1 AND 2) OR 3 7. 4 AND 5 AND 6
Embase	#1. guttapercha OR ‘gutta percha’ #2. ‘ah plus’ OR (epoxy AND resins) OR (resin AND cements) OR (epoxy AND ‘resin based’ AND root AND canal AND sealer) #3. Resilon #4. (fracture AND resistance) OR (tooth AND fracture) #5. ((# 1 AND # 2) OR # 3) AND # 4
Cochrane Library	1. MeSH Terms: Root Canal Obturation 2. MeSH Terms: Root Canal Filling Materials 3. MeSH Terms: Gutta-Percha 4. MeSH Terms: resilon sealer 5. MeSH Terms: Tooth Fractures 6. MeSH Terms: Dental Stress Analysis 7. KEY WORD: fracture resistance 8. (# 1 OR # 2 OR (# 3 AND # 4)) AND (# 5 OR # 6 OR # 7)
Web of Science	#1. TOPIC: (gutta-percha) OR TOPIC: (guttapercha) #2. TOPIC: (AH-plus) OR TOPIC: (Epoxy Resins) OR TOPIC: (Resin Cements) OR TOPIC: (epoxy resin-based root canal sealer) #3. TOPIC: (resilon) #4. TOPIC: (tooth fracture) OR TOPIC: (vertical fracture) OR TOPIC: (fracture resistance) #5. ((# 1 AND # 2) OR # 3) AND # 4
ScienceDirect	(((gutta-percha or guttapercha) AND ((AH-plus) OR ((Epoxy Resins)) OR ((Resin Cements)) OR ((epoxy resin-based root canal sealer)))) OR (resilon)) AND (((tooth fracture)) OR ((vertical fracture)) OR ((fracture resistance)))

### Literature selection criteria

The titles and abstracts of all of the retrieved articles were reviewed independently by two reviewers to determine their eligibility. If the information provided in the title and abstract was insufficient to determine the article’s relevance to this study, the full text of the article was reviewed. When there was a disagreement between the two reviewers, a discussion was held in an attempt to reach a final decision. If a final agreement could not be reached by discussion, an experienced referee was consulted. The inclusion criteria and elimination criteria are shown in Table 2.

**Table 2:** Selection Criteria

Inclusion criteria	1. Participants: Freshly extracted single rooted human teeth with closed apices, and after standard root canal preparation, all specimens had regular space for obturation.
	2. Intervention: Root canals were obturated with Gutta-percha/AH plus and (or) the Resilon system.
	3. Control: Unprepared and unfilled roots (negative control roots) or prepared but unfilled roots (positive control roots).
	4. Outcomes: Vertical fracture resistance of roots.
	5. Study design: Randomized controlled in vitro trials.
Exclusion criteria	1. Studies that did not fulfill the inclusion criteria.
	2. .Studies that evaluated the influence of different factors on the resistance to fracture of endodontically treated roots obturated with Gutta-percha/AH plus or the Resilon system.
	3. Retreatment.

### Data collection

Data in the included studies were extracted by two authors independently. These data included the first author, year of publication, sample size, tooth type, irrigation fluid, obturation technique, test machine loading rate, experimental groups and control groups.

### Assessment of risk of bias

Risk of bias of each included study was assessed by two authors based on the article’s descriptions of the following items: randomization of roots, roots free of caries or resorption, standardization of root dimensions, sample size calculation, endodontic treatment performed by a single operator, use of materials according to the instructions of the manufacturer, blinding of the examiner, and appropriateness of the statistical analyses. The category of the risk-of-bias was assessed according to the following criteria:

i. high risk of bias: one to three items were identified;
ii. medium risk of bias: four or five items were identified; and
iii. low risk of bias: six to eight items were identified.

### Data analysis

To perform the meta-analysis, the standard deviation (SD) and mean force load to VRF (expressed in Newtons) values were selected and statistically pooled using RevMan 5.3 (Cochrane Collaboration). The pooled results were expressed as standardized mean differences (SMDs) along with the 95% confidence intervals (CIs) because the outcome variable of interest was continuous. The level of statistical significance was set as *p* < 0.05. The Cochran Q test (I^2^ test) was adopted to test for heterogeneity among studies. If the heterogeneity was considerable (I^2^ > 50%), a random-effects model or subgroup analysis was used; otherwise, a fixed-effects model was employed. The reliability of the results was evaluated by performing a sensitivity analysis using Stata 12.0 software. Begg’s rank correlation test was used to evaluate publication bias when there were a sufficient number of studies included in each forest plot.

## Results

### Study search

**Fig. 1:**
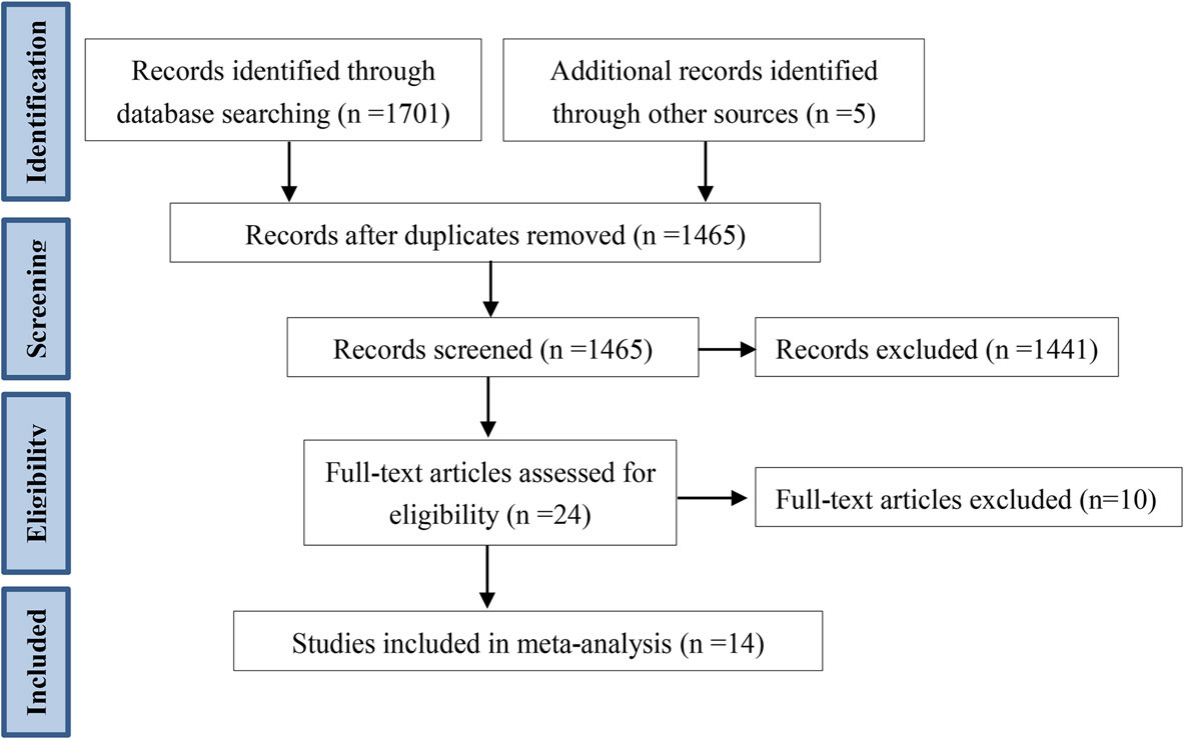
PRISMA diagram of article retrieval

The initial search yielded 1706 articles. Among them, 241 were removed as duplicates, and 1441 were excluded after reviewing the titles and abstracts. Of the remaining 24 articles, ten were excluded after careful examination of the full text. Fourteen trials [11–16, 22–29] that met the inclusion criteria were finally included. The process of literature selection is shown in Fig. 1.

### Description of studies

The studies included in this review were published from 2002 to 2017. All of the trials involved single rooted human teeth with closed apical foramen. A total of 659 roots were involved, including 92 unprepared and unfilled roots (negative control roots), 145 prepared and unfilled roots (positive control roots), 197 roots obturated with Gutta-percha/AH plus and 225 roots filled with the Resilon system. Regarding the type of tooth, human premolars were used as specimens in 10 studies [11–16, 22, 23, 25, 28], anterior teeth were used in 3 studies [26, 27, 29], and the type of tooth was not reported in 1 study [24]. Regarding the obturation techniques, a lateral condensation technique was utilized in 10 trials [13, 16, 22–29], a single-cone technique was used in 2 trials [11, 12], and no filling technique was reported in 2 trials [14, 15]. Vertical loading was applied to the teeth by the universal testing machine in all studies. The main features of the included studies are described in Table 3.

**Table 3:** Main characteristics of included studies

Author	Year	Sample size/Tooth type	Irrigants	Obturation technique	Test machine loading rate	Experimental group	Control group
Lertchirakarn et al. [27]	2002	20/Mandibular anterior teeth	EDTA and NaClO	Lateral condensation technique	0.5 mm/min	Gutta-percha/AH plus	Negative control
Hegde et al. [11]	2015	60/Mandibular premolars	EDTA and NaClO	Single-cone technique	1 mm/min	Gutta-percha/AH plus; Resilon/Epiphany	Negative control; Positive control
Topçuoğlu et al. [12]	2013	45/Mandibular premolars	EDTA and NaClO	Single-cone technique	1 mm/min	Gutta-percha/AH plus	Negative control; Positive control
Baba et al. [13]	2010	60/Mandibular premolars	EDTA and NaClO	Lateral condensation technique	1 mm/min	Gutta-percha/AH plus; Resilon/Epiphany	Positive control
Kumar et al. [15]	2014	60/Mandibular premolars	EDTA and NaClO	Not reported	1.25 mm/min	Gutta-percha/AH plus; Resilon/Realseal	Positive control
Jainaen et al. [14]	2008	40/Mandibular premolars	EDTA and NaClO	Not reported	1 mm/min	Gutta-percha/AH plus; Resilon/Realseal	Negative control; Positive control
Monteiro et al. [16]	2011	60/Mandibular premolars	EDTA and NaClO	Lateral condensation technique	1.25 mm/min	Gutta-percha/AH plus; Resilon/Realseal	Positive control
Elmakki et al. [23]	2014	60/Mandibular premolars	EDTA and NaClO	Lateral condensation technique	1 mm/min	Gutta-percha/AH plus; Resilon/Epiphany	Negative control; Positive control
Hammad et al. [24]	2007	23/Single rooted teeth	EDTA and NaClO	Lateral condensation technique	10 mm/min	Resilon/Realseal	Negative control
Nagpal et al. [28]	2012	40/Mandibular premolars	EDTA and NaClO	Lateral condensation technique	1 mm/min	Gutta-percha/AH plus; Resilon/Epiphany	Negative control
Langalia et al. [26]	2015	36/Maxillary anterior teeth	EDTA and NaClO	Lateral condensation technique	5 mm/min	Gutta-percha/AH plus; Resilon/Realseal	Negative control
Khan et al. [25]	2015	60/Mandibular premolars	EDTA and NaClO	Lateral condensation technique	1 mm/min	Resilon/Epiphany	Positive control
Dibajia et al. [22]	2017	35/Mandibular premolars	EDTA and NaClO	Lateral condensation technique	1 mm/min	Gutta-percha/AH plus; Resilon/Epiphany	Negative control
Sandikci et al. [29]	2014	60/Mandibular anterior teeth	EDTA and NaClO	Lateral condensation technique	1 mm/min	Gutta-percha/AH plus; Resilon/Epiphany	Negative control; Positive control

### Assessment of risk of bias

Of the eligible studies, eight [11, 12, 16, 22, 23, 25, 26, 29] showed a low risk of bias and six [13–15, 24, 27, 28] presented a medium risk of bias (Table 4).

**Table 4:** Risk of bias considering parameters reported in the eligible studies

Study	Teeth randomization	Teeth free of caries or resorption	Standardization of root dimensions	Sample size calculation	Endodontic treatment performed by a single operator	Materials used according to the manufacturer’s instructions	Blinding of the examiner	Appropriate of statistical analyses	Risk of bias
Lertchirakarn et al. [27]	Y	Y	N	N	Y	Y	N	Y	Medium
Hegde et al. [11]	Y	Y	Y	N	Y	Y	N	Y	Low
Topçuoğlu et al. [12]	Y	Y	Y	N	Y	Y	N	Y	Low
Baba et al. [13]	Y	N	N	N	Y	Y	N	Y	Medium
Kumar et al. [15]	Y	N	Y	N	Y	Y	N	Y	Medium
Jainaen et al. [14]	Y	Y	N	N	Y	Y	N	Y	Medium
Monteiro et al. [16]	Y	Y	Y	N	Y	Y	Y	Y	Low
Elmakki et al. [23]	Y	Y	Y	N	Y	Y	Y	Y	Low
Hammad et al. [24]	Y	Y	N	N	Y	Y	N	Y	Medium
Nagpal et al. [28]	Y	N	N	N	Y	Y	N	Y	Medium
Langalia et al. [26]	Y	Y	Y	N	Y	Y	N	Y	Low
Khan et al. [25]	Y	Y	Y	N	Y	Y	N	Y	Low
Dibaji et al. [22]	Y	Y	Y	N	Y	Y	N	Y	Low
Sandikci et al. [29]	Y	Y	Y	N	Y	Y	N	Y	Low

Y (Yes) indicates that the specific parameter was reported in the article

N (No) indicates that the specific parameter was not possible to be found in the article

### Meta-analysis

#### Gutta-percha/AH plus group verse negative control group

**Fig. 2:**
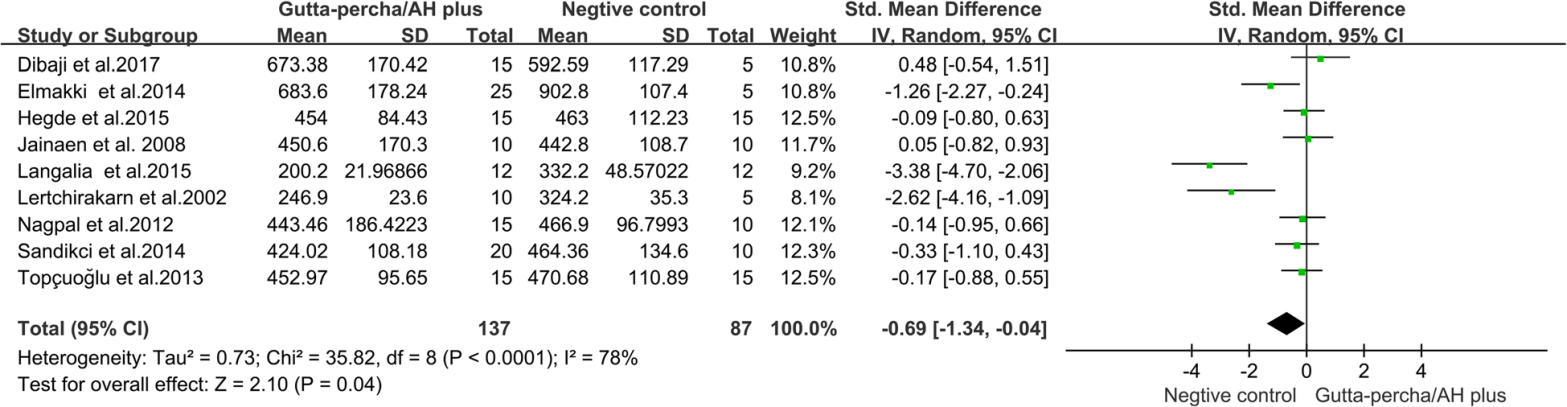
Meta-analysis of the vertical fracture resistance of roots comparing the Gutta-percha/AH plus groups with the negative control groups

Differences between a Gutta-percha/AH plus group (roots filled with Gutta-percha/AH plus) and a negative control group (unprepared and unfilled roots) were reported in nine studies [11, 12, 14, 22, 23, 26–29]. The results of the meta-analysis showed that the VRF resistance of negative control roots was significantly higher than that of roots in Gutta-percha/AH plus group (Fig. 2, SMD = − 0.69, 95% CI = − 1.34 to − 0.04, *p* = 0.04).

#### Resilon system group verse negative control group

**Fig. 3:**
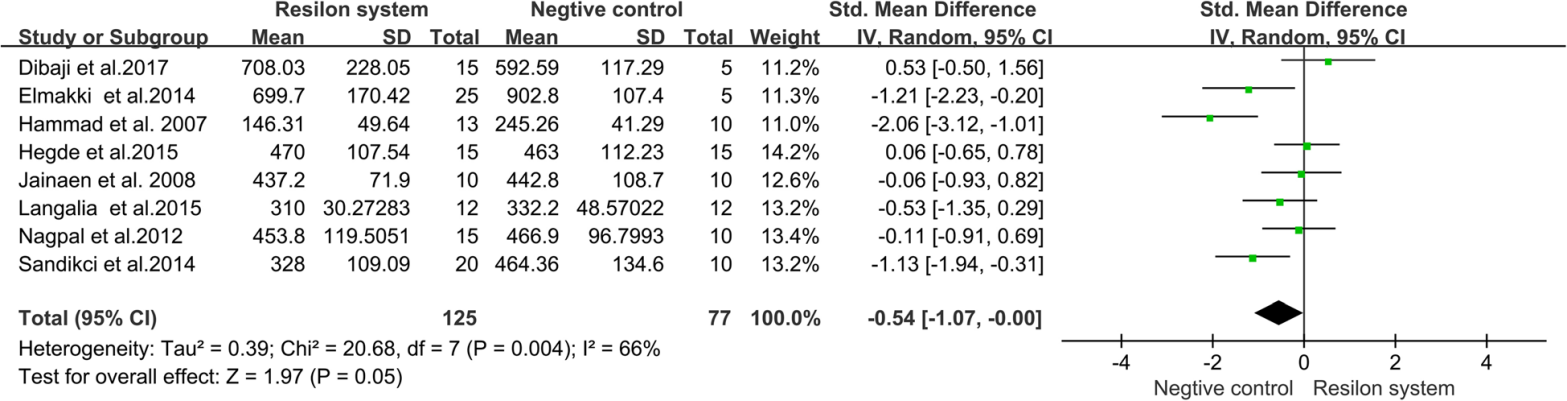
Meta-analysis of the vertical fracture resistance of roots comparing the Resilon system groups with the negative control groups

Differences between a Resilon system group (roots filled with the Resion system) and a negative control group were investigated in eight studies [11, 14, 22–24, 26, 28, 29]. The results of the meta-analysis showed that the negative control roots had higher VRF resistance than roots in Resilon system group (Fig. 3, SMD = − 0.54, 95% CI = − 1.07 to − 0.00, *p* = 0.05).

#### Gutta-percha/AH plus group verse positive control group

**Fig. 4:**
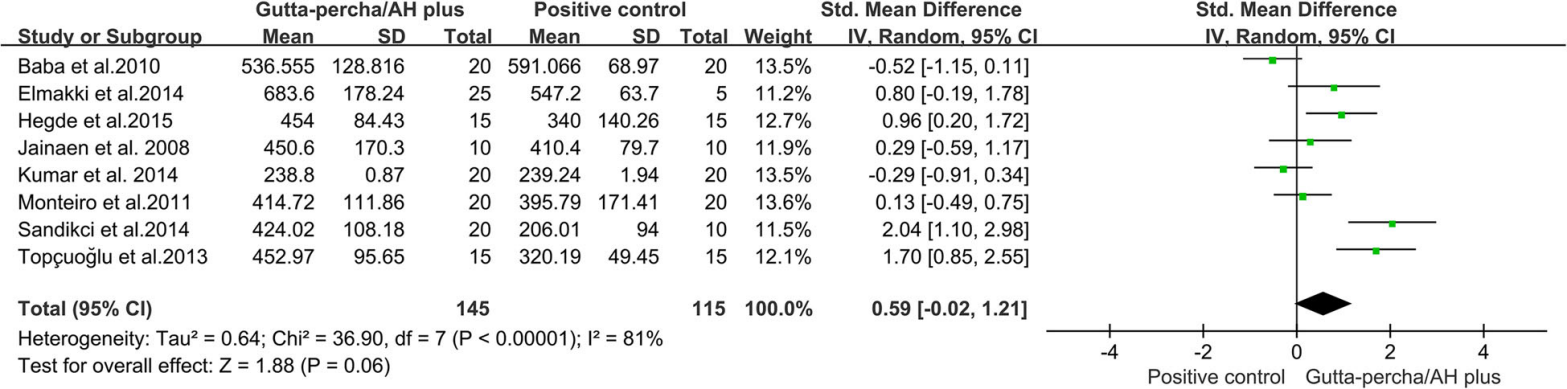
Meta-analysis of the vertical fracture resistance of roots comparing the Gutta-percha/AH plus groups with the positive control groups

Eight studies [11–16, 23, 29] reported differences in VRF resistance between a positive control group (prepared but unfilled roots) and a Gutta-percha/AH plus group. The results of the meta-analysis showed no significant difference in VRF resistance between positive control roots and roots in Gutta-percha/AH plus group (Fig. 4, SMD = 0.59, 95% CI = − 0.02 to 1.21, *p* = 0.06).

#### Resilon system group verse positive control group

**Fig. 5:**
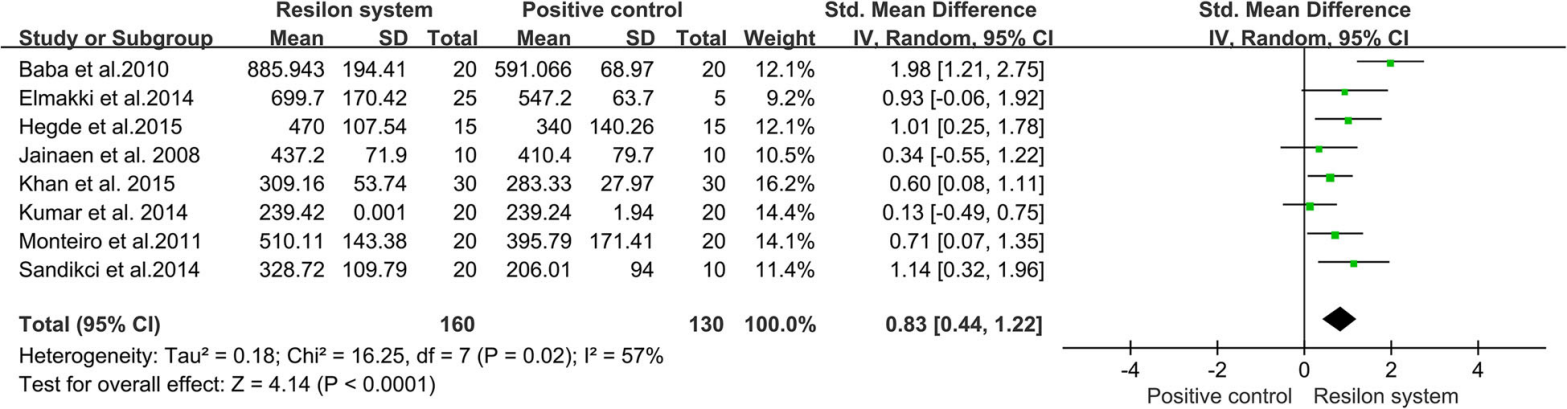
Meta-analysis of the vertical fracture resistance of roots comparing the Resilon system groups with the positive control groups

To investigate differences between a Resilon system group and a positive control group, the data from eight studies [11, 13–16, 23, 25, 29] were pooled. Analysis of the pooled data revealed that Resilon system filled roots were stronger than positive control roots (Fig. 5, SMD = 0.83, 95% CI = 0.44 to 1.22, *p* < 0.0001).

#### Gutta-percha/AH plus group verse Resilon system group

**Fig. 6:**
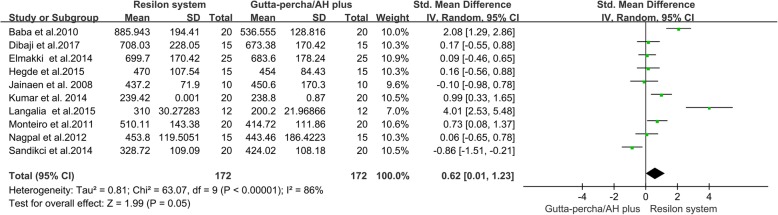
Meta-analysis of the vertical fracture resistance of roots comparing the Resilon system groups with the Gutta-percha/AH plus groups

To investigate the differences between roots filled with Resilon and roots filled with Gutta-percha/AH plus, the data from ten studies [11, 13–16, 22, 23, 26, 28, 29] were pooled. The results of the meta-analysis showed that the Resilon-filled roots had higher VRF resistance than Gutta-percha/AH plus-filled roots (Fig. 6, SMD = 0.62, 95% CI = 0.01 to 1.23, *p* = 0.05).

### Additional analysis

**Fig. 7:**
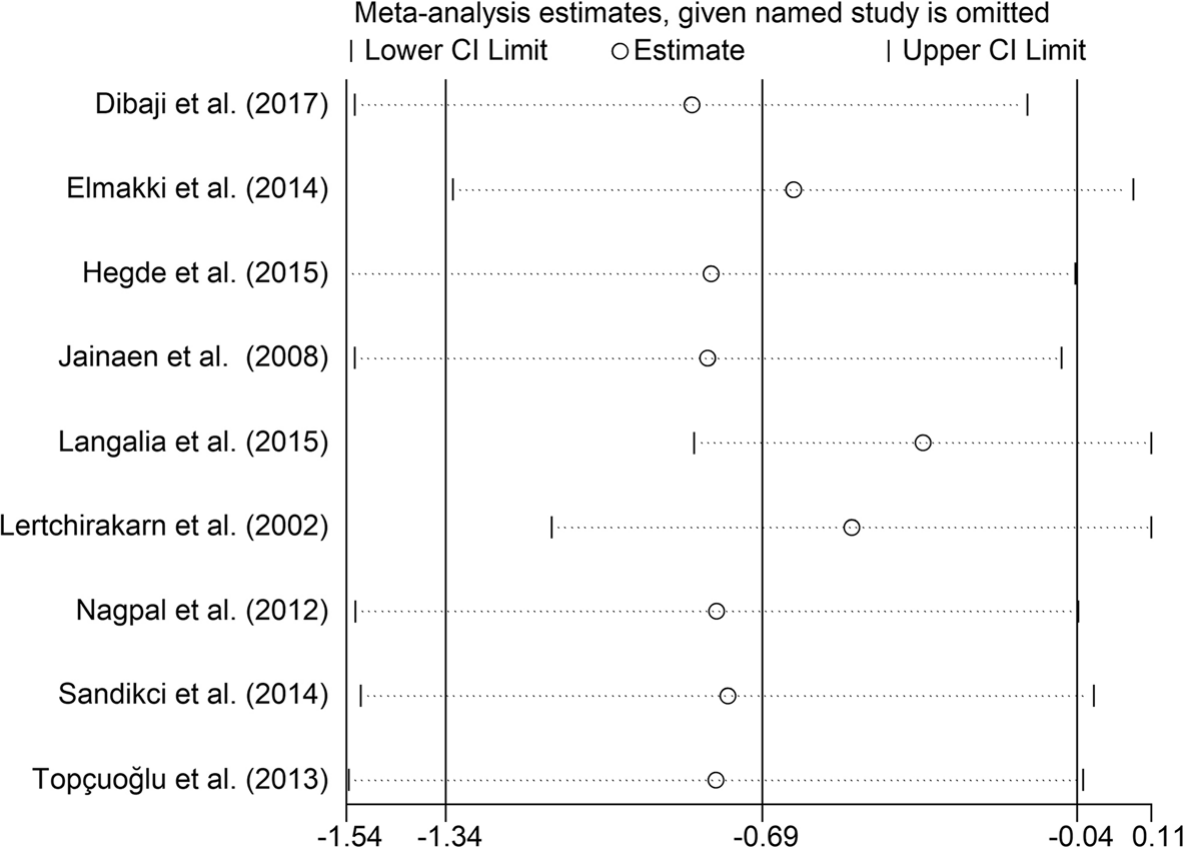
Sensitive analysis of differences between the Gutta-percha/AH plus groups and the negative control groups

**Fig. 8:**
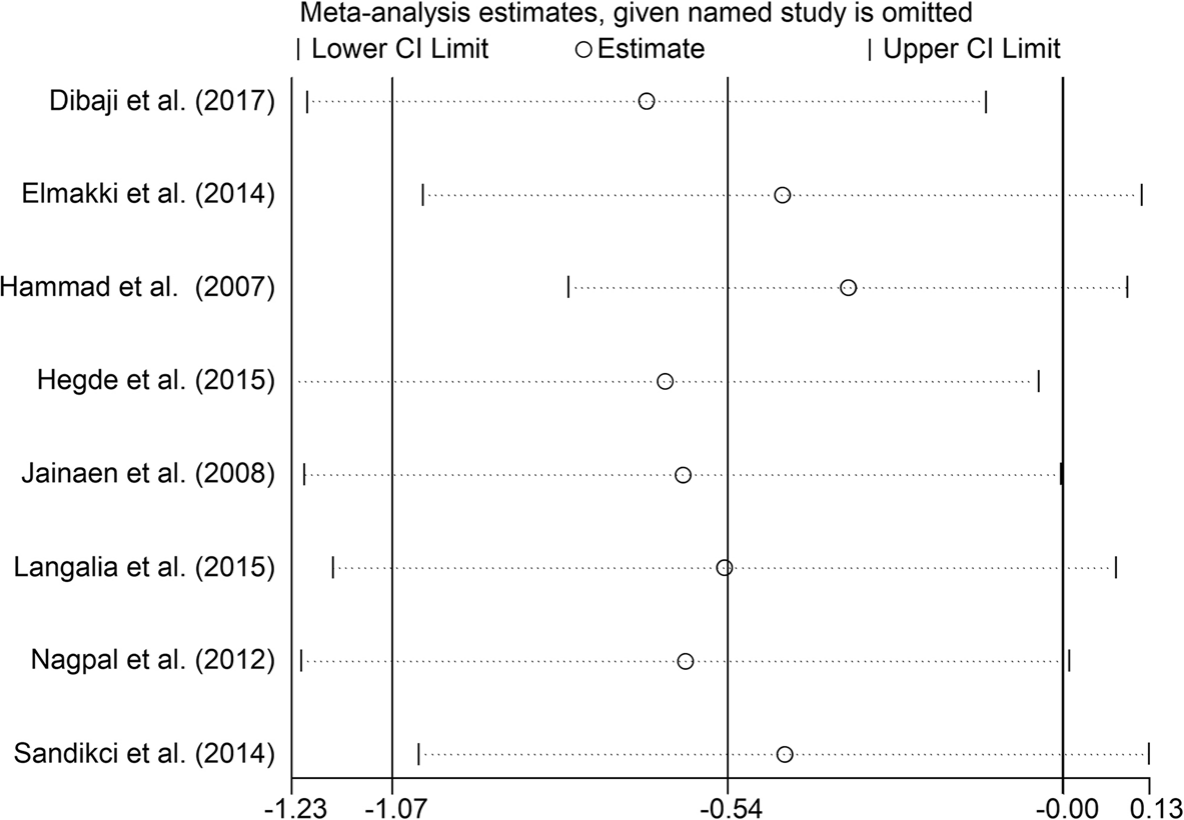
Sensitive analysis of differences between the Resilon system groups and the negative control groups

**Fig. 9:**
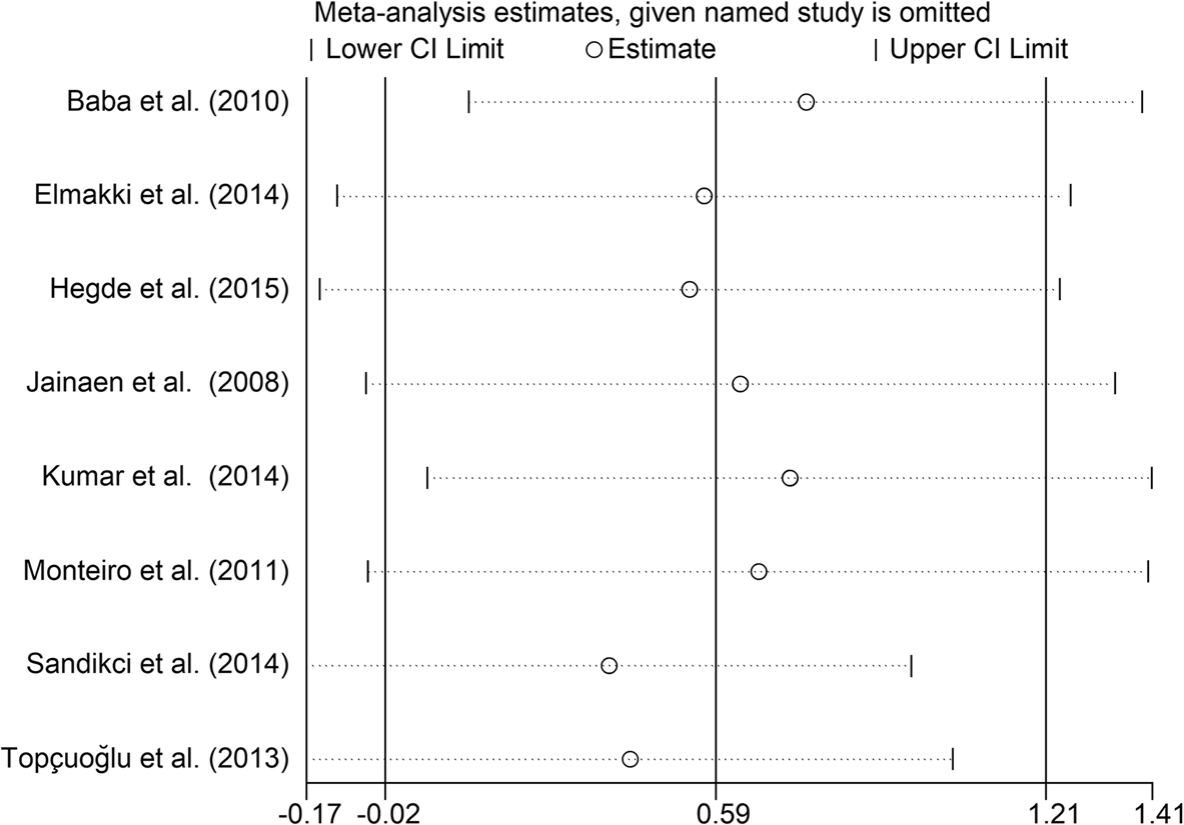
Sensitive analysis of differences between the Gutta-percha/AH plus groups and the positive control groups

**Fig. 10:**
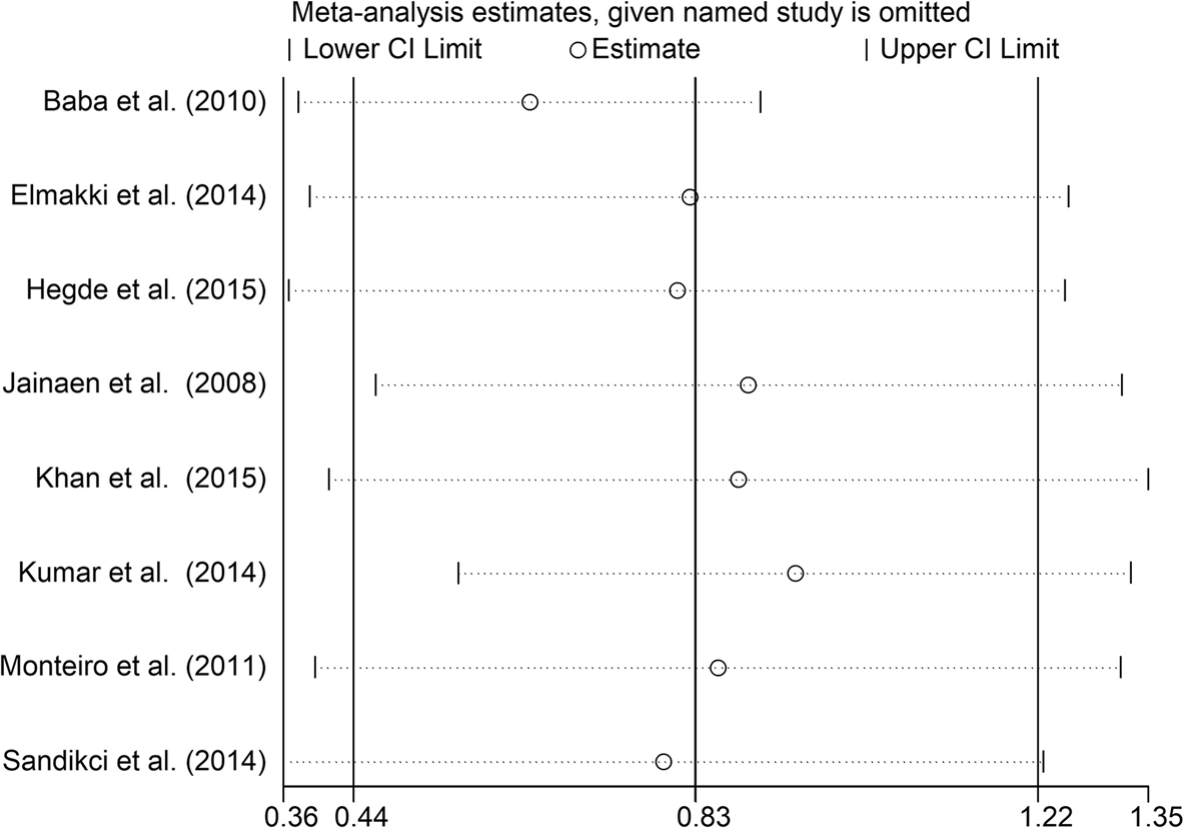
Sensitive analysis of differences between the Resilon system groups and the positive control groups

**Fig. 11:**
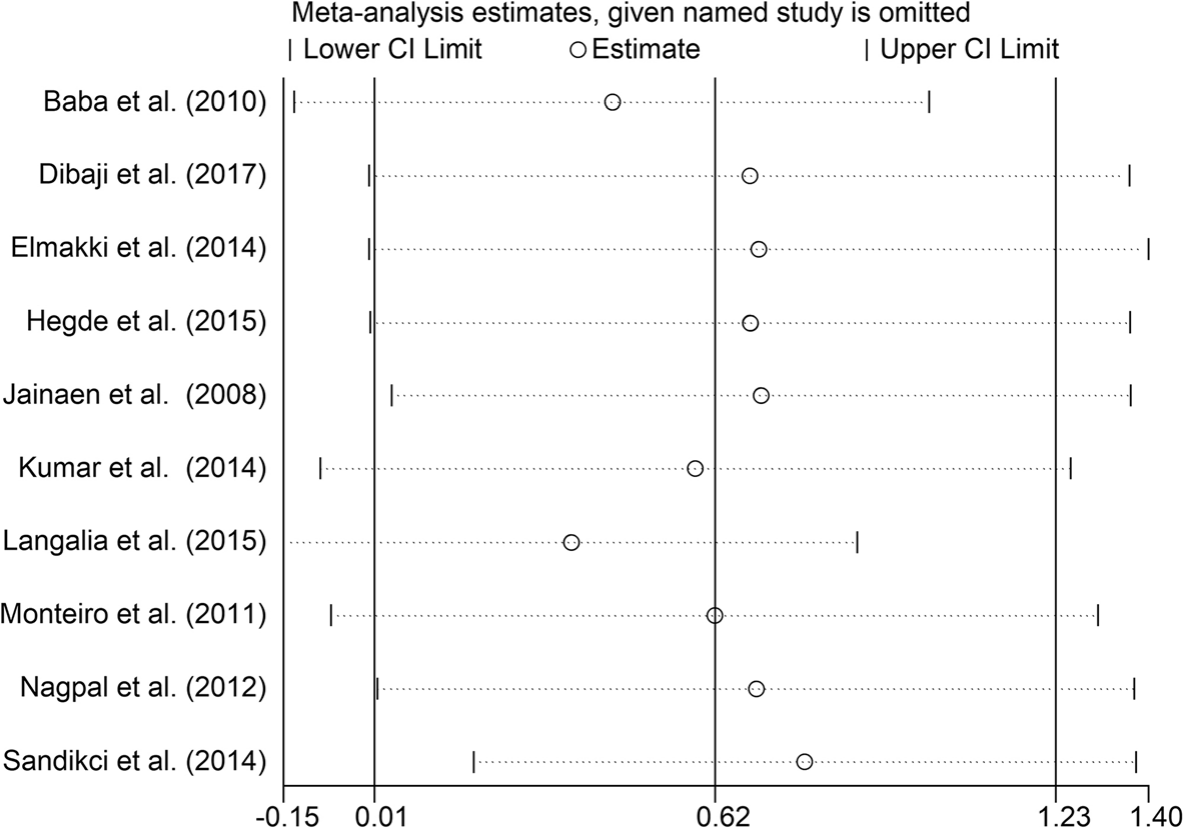
Sensitive analysis of differences between the Resilon system groups and the Gutta-percha/AH plus groups

Substantial heterogeneity existed among several of the studies. Subgroup analysis was not performed due to a lack of related information in the included studies. Efforts were made to contact their corresponding authors, but no responses were received. We conducted a sensitivity analysis to investigate the influence of a single study on the overall effect size and thereby determine the stability of the results across studies. The results remain unchanged after we omitted any single study (Figs. 7, 8, 9, 10, 11), indicating that the results are statistically stable. Publication bias could not be evaluated because of the small number of trials included in the meta-analysis.

## Discussion

VRF is often associated with the dehydration of dentin after endodontic therapy [30], removal of the root structure during root canal instrumentation [17, 19], loss of collagen cross-linking during root canal irrigation [31–34] or excessive pressure during root canal obturation [35]. At present, VRF has a high likelihood of occurring (up to 10.9%) after endodontic treatment [36]. Its occurrence typically leads to endodontic treatment failure and tooth extraction [37]. Therefore, it is important to seek an effective method to prevent VRF. Posts are typically used to reinforce endodontically treated roots [38–40]. However, their efficacy is very controversial, as it is associates with several factors which can influence the distribution of stress on the root canals and the amount of remaining dentin [41]. These factors include post type [42], length, diameter, material, and design [41], etc. If these factors are suitable, the posts might reinforce roots. If some of these factors are undesirable, the posts may play a negative role. Therefore, careful control of these factors must be taken when posts are used to reinforce endodontically treated teeth. Alternative methods to increase the VRF resistance of endodontically treated teeth have been investigated. Recently, obturating materials such as Gutta-percha and Resilon have been shown to influence the VRF resistance of root canals. However, there are different views in the literature with respect to whether these two materials can increase the postendodontic VRF resistance of roots and which one has a better reinforcement effect. These conflicting views make it difficult for clinicians to select the appropriate clinical approach. Therefore, a meta-analysis was performed to evaluate and compare the reinforcement efficacy of these two obturation systems on endodontically treated root canals. The results can offer guidance to clinicians in evidence-based decision making.

The results of this meta-analysis indicate that root canals filled with Resilon have higher fracture resistance than do prepared unfilled roots or roots filled with Gutta-percha/AH plus. These results can be attributed to the “monoblock” concept. According to Tay FR [43], a monoblock is a gap-free, solid and mechanically homogeneous mass in the root canal space that consists of different bondable materials and interfaces, which can facilitate favorable root canal sealing and simultaneously reinforce the filled canal [44, 45]. When Resilon is used to obturate a root canal, the Resilon core is bonded to the sealer (Epiphany or Realseal), and the resulting complex is bonded to the dentinal wall of the root canal [46], forming a monoblock system [47]. Resilon is a thermoplastic synthetic polymer composed of polyester with improved flexural strength. Compared with Gutta-percha, Resilon shows superior bonding potential when applied in combination with a resin-based sealer [15]. Therefore, the Resilon system has a superior ability to reinforce instrumented roots than dose the Gutta-percha/AH plus obturation system.

This meta-analysis found no significant difference in VRF resistance between prepared unfilled roots and Gutta-percha/AH plus obturated roots. Gutta-percha/AH plus has been accepted as the standard obturating system in root canal treatment. However, although the adhesive strength between the AH-plus sealer and the dentine wall is favorable [48], there is no chemical adhesion between Gutta-percha and AH-plus [8]; therefore, no monoblock system is formed, and no reinforcement is provided to the roots [49].

There was considerable heterogeneity among the included studies, which was primarily associated with methodological diversity. This diversity included differences in the type of tooth, root canal filling techniques and irrigation fluids, etc. The articles included in this meta-analysis involved different types of teeth, such as single-rooted straight maxillary anterior teeth, mandibular anterior teeth and mandibular premolars. Variation in root canal anatomies and root morphologies might affect fracture resistance of roots slightly [50]. In addition, the investigators in the eligible studies used different obturation techniques, including a lateral compaction technique and a single cone technique. The lateral compaction technique does not produce a homogeneous mass because the core material and accessory cones always remain separated, and the excessive wedge force while compacting may lead to initial root cracks [51], which might cause bias to the results of the studies with this technique and then affect the conclusion of our meta-analysis. Single cone techniques are often reliant upon sealers and may not densely obturate the canal in 3 dimensions [52], which may affect the efficacy of the obturating materials in reinforcing the roots. Furthermore, the irrigation step may influence the bonding of the obturating materials to the dentinal surface of the root. In all of the included studies, the investigators used ethylene diamine tetraacetic acid (EDTA) and sodium hypochlorite (NaClO) to remove the smear layer. However, the final irrigations differed among the studies. According to Lertchirakarn et al. [27], the high resistance of Resilon-obturated canals to fracture might be due to the clearance of the smear layer by EDTA after instrumentation which allowed the sealer to contact the canal wall and penetrate the dentinal tubules, resulting in increased root strength. In addition, it was reported that NaClO is not appropriate as the last irrigation to remove the smear layer because the residual solution may have adverse effects on the bonding strength of the primer to the dentine and may inhibit the curing of resin materials [53]. In contrast, Varela et al. [54] reported that the effect of NaClO on the polymerization of the sealer could be neglected. Due to these conflicting conclusions, the influence of final irrigation on the efficacy of the obturating materials in strengthening the roots remains unclear.

To our knowledge, this study presents the first meta-analysis performed to evaluate and compare the effects of Gutta-percha/AH plus and the Resilon system in reinforcing endodontically treated root canals. Strict inclusion and exclusion criteria were established. Only randomized controlled trials were included. Exhaustive searches of the relevant literature were performed. Fourteen studies were ultimately included. The risk of bias of these studies was strictly evaluated. Most of the included studies are well-designed research studies. A sensitivity analysis was used to explore the stability of results. The SMDs and 95% CIs did not change significantly when any one trial was removed. Therefore, our results are stable.

Although the meta-analysis was carefully conducted, some limitations remain. First, publication bias could not be evaluated because of the small number of trials included in the meta-analysis. Second, a medium risk of bias was found in some of the included studies. These studies scored especially poorly on the items including calculation of sample size and blinding of the examiner. Third, the meta-analysis is based on the findings of in vitro studies that were of low level of evidence. An in vitro approach is sometimes the only practical approach for medical or bio-medical research. However, in vitro studies have intrinsic limitations when attempting to accurately simulate biological, chemical or physical conditions in vivo [55, 56]. Although fracture resistance testing can be used to evaluate the fracture resistance of root canals filled with different materials, factors such as the temperature cycling, the wet environment, the direction of masticatory force, the frequency of loading and the presence of periodontal membrane need to be considered because they may affect the fracture resistance of roots in vivo. Therefore, the results of in vitro studies cannot be validly extrapolated to the clinical context. Even so, mechanical testing methods can offer useful information to identify substrate variables [57–60] and then provide guidance for application procedures [61, 62]. Thus, a meta-analysis based on in vitro studies is helpful to clinical practice, especially in the absence of evidence based on well-designed clinical trials [63–65]. Moreover, such a meta-analysis can suggest improvements and standardized methodologies for future studies [66, 67].

In consideration of the above results and limitations, we suggest that future randomized controlled studies perform appropriate sample size calculations, randomization and blinding, and control potentially confounding factors. Moreover, well-designed randomized controlled clinical trials are needed to evaluate the incidence of VRF of endodontically treated teeth while using these two obturating materials.

## Conclusions

In conclusion, the present study suggests that filling the canals with Gutta-percha/AH plus fails to reinforce endodontically treated root canals, whereas the Resilon obturation system can increase the VRF resistance of prepared roots. It is to be noted that the conclusion should be interpreted cautiously because this meta-analysis was based on in vitro studies.

## Abbreviations

CI: Confidence interval
EDTA: Ethylene diamine tetraacetic acid
NaClO: Sodium hypochlorite
SD: Standard deviation
SMD: Standardized mean difference
VRF: Vertical root fracture

## References

[1] Çobankara FK, Üngör M, Belli S (2002). The effect of two different root canal sealers and smear layer on resistance to root fracture. J Endod..

[2] Karapinar KM, Sunay H, Tanalp J, Bayirli G (2009). Fracture resistance of roots using different canal filling systems. Int Endond J.

[3] Lam PP, Palamara JE, Messer HH (2005). Fracture strength of tooth roots following canal preparation by hand and rotary instrumentation. Aust Endod J.

[4] D'Arcangelo C, De AF, Vadini M, D'Amario M, Caputi S (2010). Fracture resistance and deflection of pulpless anterior teeth restored with composite or porcelain veneers. J Endod..

[5] Ozcopur B, Akman S, Eskitascioglu G, Belli S (2010). The effect of different posts on fracture strength of roots with vertical fracture and re-attached fragments. J Oral Rehabil.

[6] Bortoluzzi EA, Souza EM, Reis JMSN, Esberard RM, Tanomaru-Filho M (2010). Fracture strength of bovine incisors after intra-radicular treatment with MTA in an experimental immature model. Int Endod J.

[7] Johnson ME, Stewart GP, Nielsen CJ, Hatton JF (2000). Evaluation of root reinforcement of endodontically treated teeth. Oral Surg Oral Med Oral Pathol Oral Radiol Endod..

[8] Teixeira FB, Teixeira ECN, Thompson JY, Trope M (2004). Fracture resistance of roots endodontically treated with a new resin filling material. J Am Dent Assoc.

[9] Pascon EA, Spangberg LS (1990). In vitro cytotoxicity of root canal filling materials: 1. Gutta-percha. J Endod..

[10] Bhat SS, Hegde SK, Rao A, Shaji Mohammed AK (2012). Evaluation of resistance of teeth subjected to fracture after endodontic treatment using different root canal sealers: an in vitro study. J Indian Soc Pedod Prev Dent.

[11] Hegde V, Arora S (2015). Fracture resistance of roots obturated with novel hydrophilic obturation systems. J Conserv Dent.

[12] Topçuoğlu HS, Tuncay Ö, Karataş E, Arslan H, Yeter K (2013). In vitro fracture resistance of roots obturated with epoxy resin-based, mineral trioxide aggregate-based. and bioceramic root canal sealers J Endod.

[13] Baba SM, Grover SI, Tyagi V (2010). Fracture resistance of teeth obturated with Gutta percha and Resilon: an in vitro study. J Conserv Dent.

[14] Jainaen A, Palamara JE, Messer HH (2009). The effect of resin-based sealers on fracture properties of dentine. Int Endod J.

[15] Kumar P, Kaur NM, Arora S, Dixit S (2014). Evaluation of fracture resistance of roots obturated with resilon and thermoplasticized gutta-percha: an in vitro study. J Conserv Dent.

[16] Monteiro J (2011). de Noronha de Ataide I, Chalakkal P, Chandra PK. In vitro resistance to fracture of roots obturated with Resilon or Gutta-percha. J Endod..

[17] Schäfer E, Zandbiglari T, Schäfer J (2007). Influence of resin-based adhesive root canal fillings on the resistance to fracture of endodontically treated roots: an in vitro preliminary study. Oral Surg Oral Med Oral Pathol Oral Radiol Endod.

[18] Zamin C, Silva-Sousa YTC, Souza-Gabriel AE, Messias DF, Sousa-Neto MD (2012). Fracture susceptibility of endodontically treated teeth. Dent Traumatol.

[19] Zandbiglari T, Davids H, Schäfer E (2006). Influence of instrument taper on the resistance to fracture of endodontically treated roots. Oral Surg Oral Med Oral Pathol Oral Radiol Endod..

[20] Mohammadi Z, Jafarzadeh H, Shalavi S, Bhandi S, Kinoshita J (2015). Resilon: review of a new material for obturation of the canal. J Contemp Dent Pract.

[21] Soares C, Maia C, Vale F, Gadêneto C, Carvalho L, Oliveira H (2015). Comparison of endodontic retreatment in teeth obturated with Resilon or Gutta-Percha: a review of literature. Iran Endod J..

[22] Dibaji F, Afkhami F, Bidkhori B, Kharazifard MJ (2017). Fracture resistance of roots after application of different sealers. Iran Endod J.

[23] Elmakki F, Abu-bakr N, Ibrahim Y (2013). Fracture resistance of Resilon/epiphany and Gutta percha/AH plus. Indian J Dent.

[24] Hammad M, Qualtrough A, Silikas N (2007). Effect of new obturating materials on vertical root fracture resistance of endodontically treated teeth. J Endod..

[25] Khan S, Inamdar MN, Munaga S, Ali SA, Rawtiya M, Ahmad E (2015). Evaluation of fracture resistance of endodontically treated teeth filled with Gutta-Percha and Resilon obturating material: an in vitro study. J Int Oral Health..

[26] Langalia AK, Dave B, Patel N, Thakkar V, Sheth S, Parekh V (2015). Comparative evaluation of fracture resistance of endodontically treated teeth obturated with resin based adhesive sealers with conventional obturation technique: an in vitro study. J Int Oral Health.

[27] Lertchirakarn V, Timyam A, Messer HH (2002). Effects of root canal sealers on vertical root fracture resistance of endodontically treated teeth. J Endod..

[28] Nagpal A, Annapoorna BM, Prashanth MB, Prashanth NT, Singla M, Deepak BS (2012). A comparative evaluation of the vertical root fracture resistance of endodontically treated teeth using different root canal sealers: an in vitro study. J Contemp Dent Pract.

[29] Sandikci T, Kaptan RF (2014). Comparative evaluation of the fracture resistances of endodontically treated teeth filled using five different root canal filling systems. Niger J Clin Pract.

[30] Helfer AR, Melnick S, Schilder H (1972). Determination of the moisture content of vital and pulpless teeth. Oral Surg Oral Med Oral Pathol Oral Radiol.

[31] Ari H, Erdemir A, Belli S (2004). Evaluation of the effect of endodontic irrigation solutions on the microhardness and the roughness of root canal dentin. J Endod..

[32] Cruz-Filho AM, Sousa-Neto MD, Saquy PC, Pécora JD (2001). Evaluation of the effect of EDTAC, CDTA, and EGTA on radicular dentin microhardness. J Endod.

[33] Rivera EM, Yamauchi M (1993). Site comparisons of dentine collagen cross-links from extracted human teeth. Arch Oral Biol.

[34] Slutzkygoldberg I, Maree M, Liberman R, Heling I (2004). Effect of sodium hypochlorite on dentin microhardness. J Endod..

[35] Holcomb JQ, Pitts DL, Nicholls JI (1987). Further investigation of spreader loads required to cause vertical root fracture during lateral condensation. J Endod..

[36] Fuss Z, Lustig J, Tamse A (1999). Prevalence of vertical root fractures in extracted endodontically treated teeth. Int Endod J.

[37] Tsesis I, Rosen E, Tamse A, Taschieri S, Kfir A (2010). Diagnosis of vertical root fractures in endodontically treated teeth based on clinical and radiographic indices: a systematic review. J Endod..

[38] Assif D, Gorfil C (1994). Biomechanical considerations in restoring endodontically treated teeth. J Prosthet Dent.

[39] Cohen BI, Pagnillo M, Condos S, Deutsch AS (1995). Comparison of the torsional forces at failure for seven endodontic post systems. J Prosthet Dent.

[40] Gutmann JL (1992). The dentin-root complex: anatomic and biologic considerations in restoring endodontically treated teeth. J Prosthet Dent.

[41] Fernandes AS, Dessai GS (2001). Factors affecting the fracture resistance of post-core reconstructed teeth: a review. Int J Prosthodont.

[42] Testori T, Badino M, Castagnola M (1993). Vertical root fractures in endodontically treated teeth: a clinical survey of 36 cases. J Endod..

[43] Tay FR, Pashley DH (2007). Monoblocks in root canals: a hypothetical or a tangible goal. J Endod..

[44] Schwartz RS (2006). Adhesive dentistry and endodontics. Part 2: bonding in the root canal system-the promise and the problems: a review. J Endod..

[45] Teixeira FB, Teixeira EC, Thompson J, Leinfelder KF, Trope M (2004). Dentinal bonding reaches the root canal system. J Esthet Restor Dent.

[46] Shipper G, D Ø, Teixeira FB, Trope M (2004). An evaluation of microbial leakage in roots filled with a thermoplastic synthetic polymer-based root canal filling material (Resilon). J Endod..

[47] Belli S, Eraslan O, Eskitascioglu G, Karbhari V (2011). Monoblocks in root canals: a finite elemental stress analysis study. Int Endod J.

[48] Sousa-Neto MD, Marchesan MA, Pécora JD, Junior AB, Silva-Sousa YT, Saquy PC (2002). Effect of Er:YAG laser on adhesion of root canal sealers. J Endod..

[49] Aptekar A, Ginnan K (2006). Comparative analysis of microleakage and seal for 2 obturation materials: Resilon/epiphany and gutta-percha. J Can Dent Assoc.

[50] Obermayr G, Walton RE, Leary JM, Krell KV (1991). Vertical root fracture and relative deformation during obturation and post cementation. J Prosthet Dent.

[51] Meister F, Lommel TJ, Gerstein H (1980). Diagnosis and possible causes of vertical root fractures. Oral Surg Oral Med Oral Pathol.

[52] Whitworth J (2005). Methods of filling root canals: principles and practices. Endod Top.

[53] Darcey J, Roudsari RV, Jawad S, Taylor C, Hunter M (2016). Modern endodontic principles. Part 5: obturation. Dent Update.

[54] Varela SG, Rábade LB, Lombardero PR, Sixto JM, Bahillo JD, Park SA (2003). In vitro study of endodontic post cementation protocols that use resin cements. J Prosthet Dent.

[55] Sarkisonofre R, Skupien JA, Cenci MS, Moraes RR, Pereiracenci T (2014). The role of resin cement on bond strength of glass-fiber posts luted into root canals: a systematic review and meta-analysis of in vitro studies. Oper Dent.

[56] Masarwa N, Mohamed A, Abourabii I, Abu ZR, Steier L (2016). Longevity of self-etch dentin bonding adhesives compared to etch-and-rinse dentin bonding adhesives: a systematic review. J Evid Based Dent Pract.

[57] Frankenberger R, Krämer N, Lohbauer U, Nikolaenko SA, Reich SM (2007). Marginal integrity: is the clinical performance of bonded restorations predictable in vitro?. J Adhes Dent.

[58] Hebling J, Castro FL, Costa CA (2007). Adhesive performance of dentin bonding agents applied in vivo and in vitro. Effect of intrapulpal pressure and dentin depth. J Biomed Mater Res B Appl Biomater.

[59] Shono Y, Ogawa T, Terashita M, Carvalho RM, Pashley EL, Pashley DH (1999). Regional measurement of resin-dentin bonding as an array. J Dent Res.

[60] Shono Y, Terashita M, Pashley EL, Brewer PD, Pashley DH (1997). Effects of cross-sectional area on resin-enamel tensile bond strength. Dent Mater.

[61] Braga RR, Meira JBC, Boaro LCC, Xavier TA (2010). Adhesion to tooth structure: a critical review of “macro” test methods. Dent Mater.

[62] Hashimoto M, Tay FR, Svizero NR, de Gee AJ, Feilzer AJ, Sano H (2007). The effects of common errors on sealing ability of total-etch adhesives. Dent Mater.

[63] Nassar U, Aziz T, Flores-Mir C (2011). Dimensional stability of irreversible hydrocolloid impression materials as a function of pouring time: a systematic review. J Prosthet Dent.

[64] West NX, Davies M, Amaechi BT (2011). In vitro and in situ Erosion models for evaluating tooth substance loss. Caries Res.

[65] Bayne SC (2012). Correlation of clinical performance with ‘in vitro tests’ of restorative dental materials that use polymer-based matrices. Dent Mater.

[66] Greenhalgh T (1997). How to read a paper: papers that summarise other papers (systematic reviews and meta-analyses). BMJ.

[67] Linde K, Willich SN (2003). How objective are systematic reviews? Differences between reviews on complementary medicine. J R Soc Med.

